# Camel whey protein enhances lymphocyte survival by modulating the expression of survivin, bim/bax, and cytochrome C and restores heat stress-mediated pathological alteration in lymphoid organs

**DOI:** 10.22038/IJBMS.2018.27584.6729

**Published:** 2018-09

**Authors:** Nancy K Ramadan, Gamal Badr, Hanem S Abdel-Tawab, Samia F Ahmed, Mohamed H Mahmoud

**Affiliations:** 1 Zoology Department, Faculty of Science, Assiut University, 71516 Assiut, Egypt; 2 Animal Health Research Institute, Assiut Branch, 71526 Assiut, Egypt; 3 Laboratory of Immunology & Molecular Physiology, Zoology Department, Faculty of Science, Assiut University, 71516 Assiut, Egypt; 4 Deanship of Scientific Research, King Saud University, Riyadh, Saudi Arabia; 5 Food Science and Nutrition Department, National Research Center, Dokki, 12622 Cairo, Egypt

**Keywords:** Antioxidants, Apoptosis, Camel whey protein, Free radicals, Heat stress

## Abstract

**Objective(s):**

Heat stress (HS) is a catastrophic stressor that dampens immunity. The current study investigates the effect of dietary administration with camel whey protein (CWP) on apoptotic pathway caused by HS.

**Materials and Methods:**

Forty-five male mice were divided into three groups: a control group; HS group; and HS mice that were orally supplemented with CWP (CWP-HS group).

**Results:**

We found that reactive oxygen species (ROS), pro-inflammatory cytokines (IL-6), and C reactive protein (CRP) were elevated in the HS group along with a significant increase of caspase-9 and -3 and decrease of total antioxidant capacity (TAC). HS mice revealed impaired phosphorylation of Bcl-2 and Survivin, as well as increased expression of Bax, Bim and cytochrome C. Additionally, we observed an aberrant distribution of HSP-70 expressing lymphocytes in the spleen and thymus of HS mice. Moreover, histopathological examination showed alterations on the architectures of immune organs. In comparison with CWP-HS group, we found that CWP restored the levels of ROS, IL-6, TAC and CRP induced by HS. Furthermore, CWP restored the expression of Bcl-2/Bax, improved the histopathological changes in immune organs and HSP-70 distribution in the spleen and thymus.

**Conclusion:**

Our findings revealed the possible ameliorative role of CWP supplementation against damages induced by exposure to HS.

## Introduction

Heat stress (HS) is one of the most serious stressors in tropical areas, which occurs when the ambient temperature is over the animal thermoneutral zone (comfort zone) (1). HS suppresses animal health, milk production and reproduction (2). HS also causes serious economic problems (3). During the transition period, heat-stressed cows suffer from depressed feed intake, negative energy balance (4), oxidative stress (5), and compromised immunity (6). Calves born to heat-stressed cows were found to suffer from compromised cellular immunity and passive immune transfer (7). Animal response to HS is a complex process involving physiological alterations (8), gene expression (9), and immune response (10). Heat shock protein 70 (HSP-70) is highly expressed as a consequence of HS, and it helps in folding and refolding of damaged proteins (11). HSP-70 maintains homeostasis of the intracellular proteins and prevents the formation of toxic aggregates that lead to inflammation or cell death (12). The best management strategies used to overcome adverse effects of HS include physical modification such as cooling and shading, genetic modification of heat-tolerant breeds, and nutritional supplementation (13). Nutritional supplementation helps to enhance the animal antioxidant capacity such as vitamins and minerals (14). Indeed, camel whey protein (CWP) contains lactoferrin (LF), serum albumin (SA), α–lactalbumin (α-LA) and different immunoglobulins (Ig) (15-17). Camel colostral whey is rich in SA, also contains abundant amounts of α-LA and LF (18). CWP exerts many immune functions, such as lymphocyte activation and proliferation, cytokine secretion, and stimulate natural killer cell activity (19-21). Additionally, whey peptides could modulate the immune response such as increasing phagocytosis, stimulating lymphocytes, and the secretion of immunoglobulin A (IgA) from Peyer’s patches (20). The antioxidant activity of WP is mainly due to its role in glutathione synthesis (22-24). There are two main apoptotic pathways: the mitochondrial pathway (intrinsic) and the death receptor pathway (extrinsic) (25). In the death receptor pathway, the receptors bind to their specific death ligands leading to the activation of caspase cascade (26). In the mitochondrial pathway, apoptosis is triggered via the pro-apoptotic signals such as Bax and Bim, which are members of the Bcl-2 family. Bcl-2 family members are classified into pro-apoptotic members such as Bax and Bim, and anti-apoptotic members such as Bcl-2 and Bcl-XL (27). During apoptosis, Bax enters to mitochondria to enhance the opening of the mitochondrial ion voltage channel thus increasing mitochondrial membrane permeability. Then, cytochrome C exits mitochondria through these ion channels and binds to Apaf 1, leading to activation of pro-caspase-9 and caspase-3, where caspase-3 accomplishes the cellular apoptosis (28). Caspase-3 is involved in both apoptotic pathways and it is the final executor of apoptosis and the most effective one in the apoptotic pathways (29). However, Bcl-2 has an opposite role as anti-apoptotic agent, and it suppresses the release of cytochrome C to the cytoplasm (30). To regulate apoptosis, Bax and Bcl-2 form homodimer to enhance apoptosis or heterodimer to suppress apoptosis (31). Even the ratio of Bax to Bcl-2 would be the molecular switch of the process of apoptosis (25, 32). 

However, there are no studies about the impact of CWP on HS in an animal model. Therefore, the current study focused on explaining the mechanism by which CWP improves impaired immune function due to exposure to HS.

## Materials and Methods

***Preparation of camel whey proteins***

We prepared CWP as previously described (21, 33). The prepared CWPs were freeze-dried and refrigerated until further use. 

***Ethics declarations***

All the experiments that were carried out on the mice in this study was reviewed and approved by the Ethics Committee at the Faculty of Medicine, Assiut University, Egypt (Approval number is IR800008718), in accordance with the Helsinki Declaration. 

***Experimental animal model***

Forty five male Balb/C mice (25–30 g) were obtained from Theodor Bilharz Research Institute, Cairo, Egypt. In the present study, we made efforts to minimize animal distress. Before the experiment, the animals were allowed to acclimate for seven days and were then housed in metal cages in a well-ventilated animal house in Faculty of Science. The animals were then maintained under standard laboratory conditions (23°C, 60-70% relative humidity and a 12-hr light/dark cycle), fed a standard diet of commercial pellets and given water *ad libitum* as previously described (34, 35). For induction of HS, thirty mice were placed in a hot chamber with a temperature of 40°C for 2 hr daily for one month, in a similar way to that previously described (36). Animals were divided into three experimental groups (n = 15/group) as following: control group, mice that were orally supplemented with distilled water (250 µl/mouse/day for one month by oral gavage); HS group, heat-stressed mice that were orally supplemented with distilled water (250 µl/mouse/day for one month by oral gavage); and CWP-HS group, heat-stressed mice that were orally supplemented with CWP (200 mg/kg body weight dissolved in 250 µl distilled water/day for one month through oral gavage). We determined the optimal dose of CWP on the basis of its LD50 and various established parameters.

**Figure 1:**
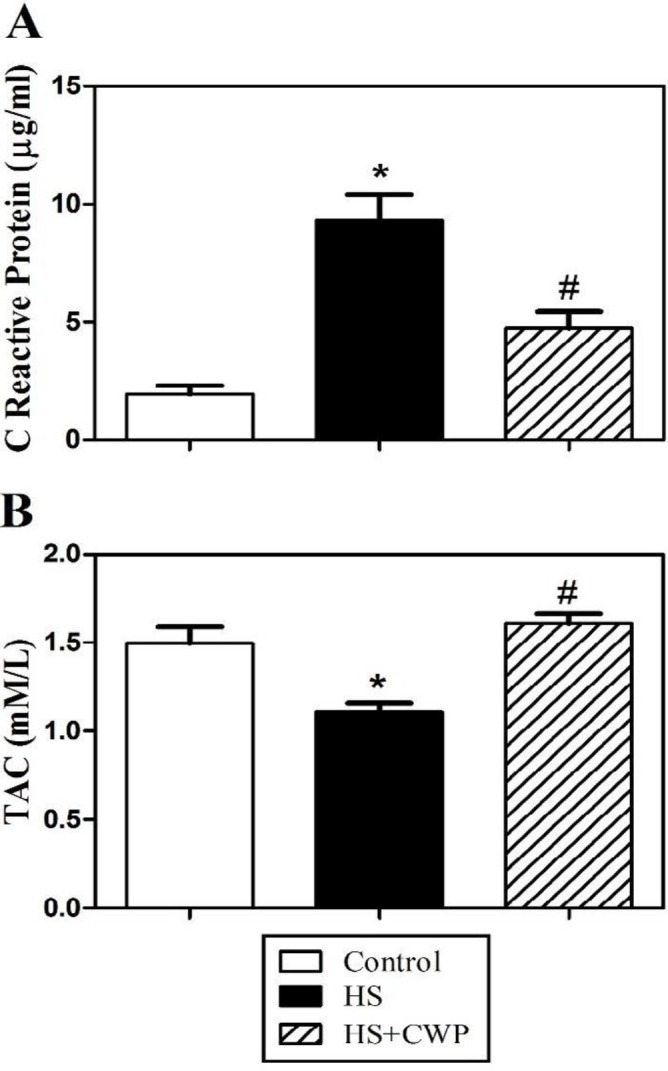
Influence of heat stress (HS) and camel whey protein (CWP) on total antioxidant capacity (TAC) and C reactive protein (CRP) in plasma. AC and CRP were measured in the plasma of HS (closed black bars) and CWP-HS (hatched bars) mice to determine the destructive effect of HS and the improving effect of CWP as compared to control mice (open bars). (A) TAC values are presented as (mM/l). (B) CRP values are presented as (µg/ml). Data from five mice per group are expressed as means ± SEM. The data were analyzed by ANOVA with Tukey’s *post hoc* test. Differences were considered statistically significant at *P<0.05 for HS vs. control; +*P*<0.05 for CWP-HS vs. control; and #*P*<0.05 CWP- HS vs. HS

**Figure 2:**
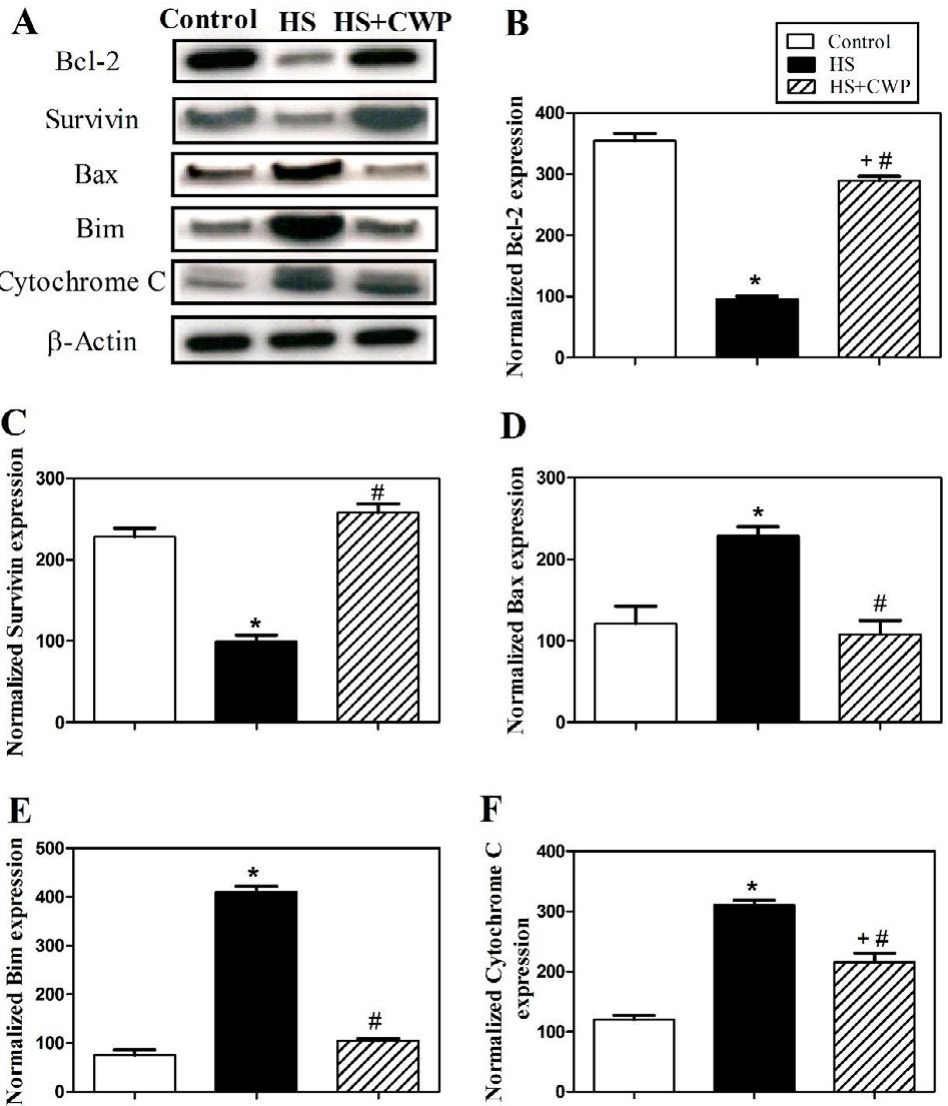
Heat stress (HS) and camel whey protein (CWP) impact on the expression of Bcl-2 family members and on the expression of Survivin. (A) Western blotting analysis were performed on white blood cell lysates of control, HS and CWP-HS animal groups using anti-Bcl-2, anti-Survivin, anti-Bax, anti-Bim, and anti-cytochrome C antibodies. The protein bands from one representative experiment are shown, and the expression of all indicated proteins was normalized to total β–actin protein levels. Data from five mice per group are expressed as the mean expression values ± SEM for (B) Bcl-2; (C) Survivin; (D) Bax; (E) Bim; and (F) cytochrome C. The data were analyzed by ANOVA with Tukey’s *post hoc *test. Differences were considered statistically significant at *P<0.05 for HS vs. control; +*P*<0.05 for CWP-HS vs. control; and #*P*<0.05 CWP- HS vs. HS

**Figure 3:**
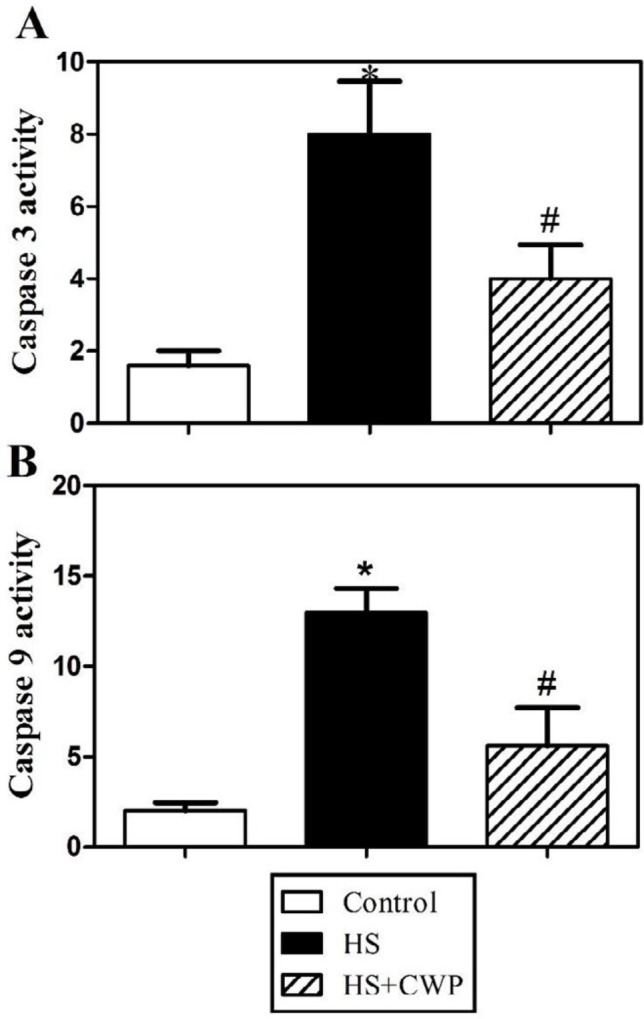
The opposite effects of heat stress (HS) and camel whey protein (CWP) on caspases activity. Accumulated data from five mice per group are expressed as mean ± SEM for (A) caspase-3 values; and (B) caspase-9 values of control (open bars), HS (closed black bars), and CWP-HS (hatched bars) animals. The data were analyzed by ANOVA with Tukey’s *post hoc* test. Differences were considered statistically significant at *P<0.05 for HS vs. control; +*P*<0.05 for CWP-HS vs. control; and #*P*<0.05 CWP- HS vs. HS

**Figure 4:**
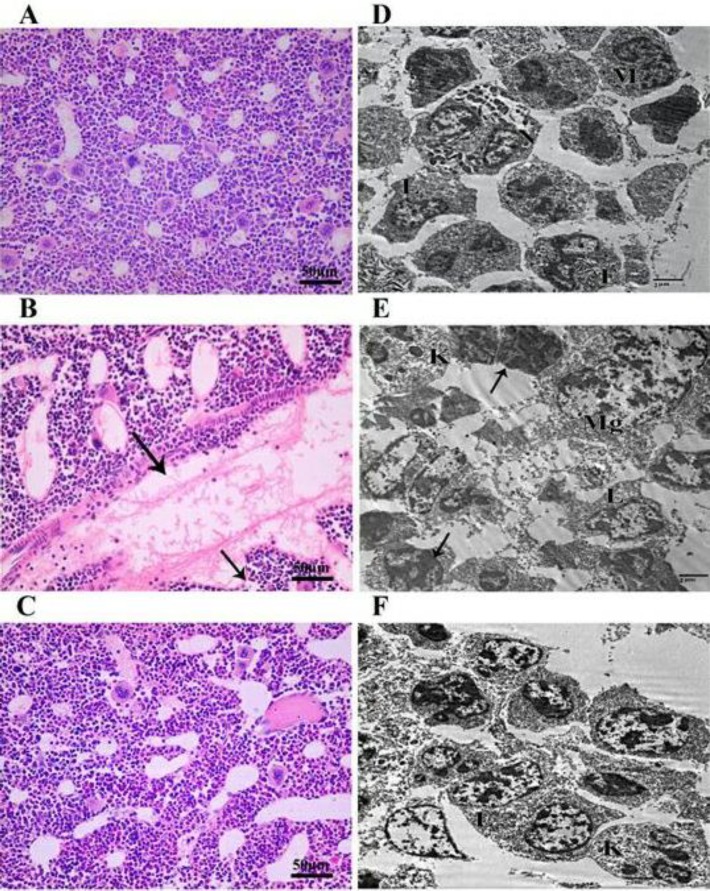
Impact of heat stress (HS) and camel whey protein (CWP) on hematopoietic system of bone marrow as a primary lymphoid organ. Sections of bone marrow from control, HS, and CWP-HS mice stained with H&E (A-C). Transmission electron microscope (TEM) showing different bone marrow cells in the same 3 animals groups (D-F). Neutrophil (N), Eosinophil (E), Monocyte (M), Lymphocyte (L), karyorrhexis (K), and Megakaryocyte (Mg)

**Figure 5:**
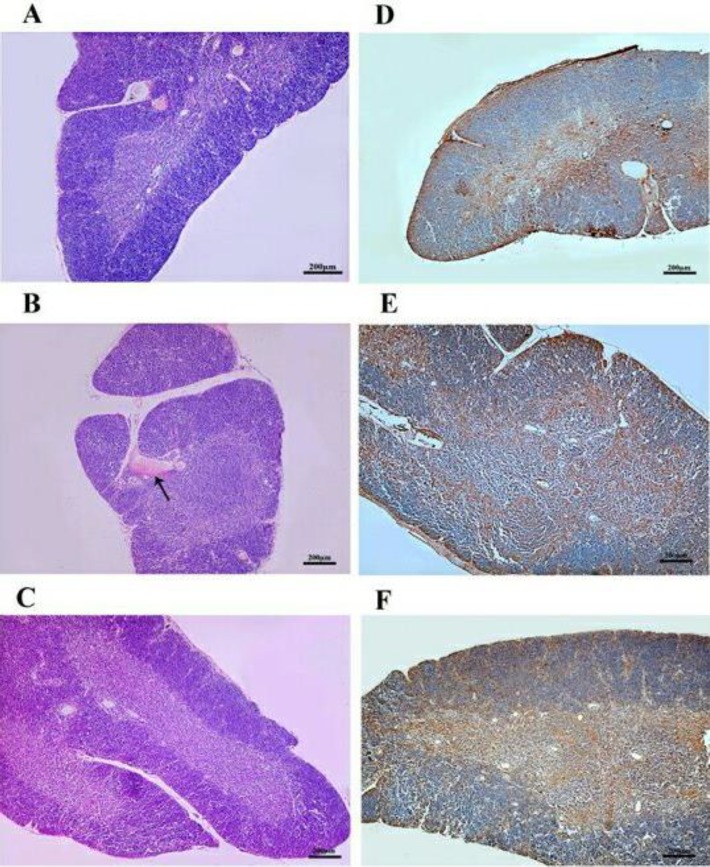
Heat stress (HS) and camel whey protein (CWP) altered effects on the histology of thymus and distribution of heat shock protein-70 (HSP-70) in thymocytes. Histological sections of thymus gland of the three animal groups were stained with H&E (A-C). Similar additional sections were immunohistochemically stained with anti-HSP-70 (D-F)

**Figure 6:**
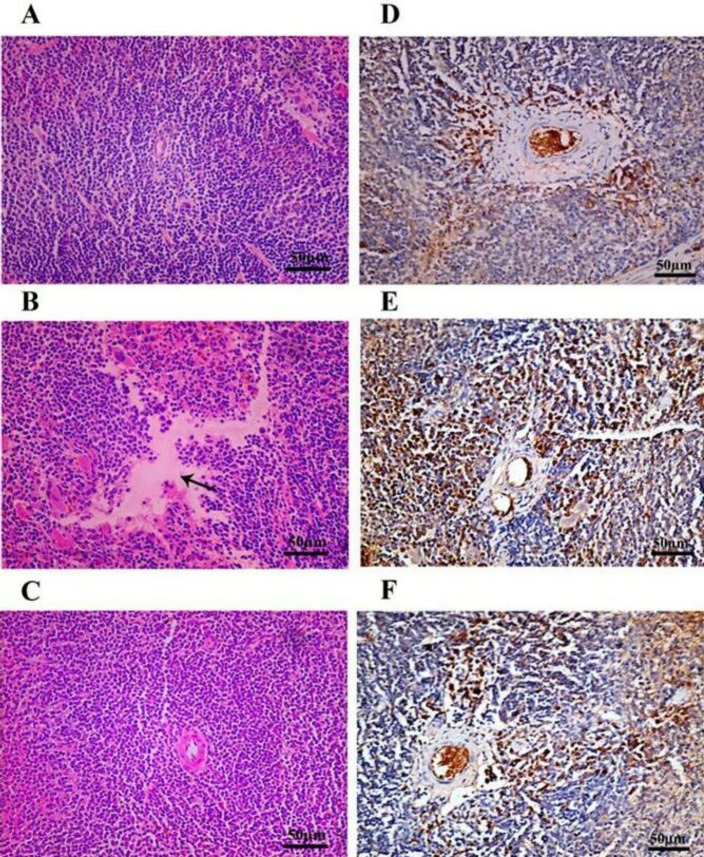
Influence of heat stress (HS) and camel whey protein (CWP) on spleen architecture and on heat shock protein-70 (HSP-70) pattern in spleen lymphocytes. Spleen sections from the three animal groups were stained with H&E (A-C). These sections were also immunohistochemically stained with antibody recognizing HSP-70 (D-F) for the same 3 animals groups

**Table 1:** The effects of heat stress (HS) and camel whey protein (CWP) on some biochemical parameters

**Parameters**	**IL-6 (pg/ml)**	**ROS (nmol/ml)**
**Control mice**	64.10 ± 2.193	34.80 ± 0.9359
**HS mice**	141.0 ± 3.232 *	64.76 ± 2.083 *
**HS+CWP mice**	79.18 ± 4.977#+	39.06 ± 1.712#

The levels of IL-6 and reactive oxygen species (ROS) were measured by ELISA. IL-6 values are presented as (pg/ml) and ROS values are presented as (nmol/ml). The results from five mice per group are expressed as the mean level of each parameter ± SEM. The data were analyzed by ANOVA with Tukey’s *post hoc *test. Differences were considered statistically significant at *P<0.05 for HS vs. control;

* P < 0.05 HS vs. control

+ P < 0.05 HS+CWP vs. control

# P < 0.05 HS+CWP vs. HS

***Quantification of blood biochemical parameters***

Following one month of supplementation with CWP or distilled water, all animals were anesthetized using pentobarbital (60 mg/kg body weight), whole blood samples were collected from the abdominal aorta, and immediately transferred into EDTA tubes. Heparinized blood was centrifuged at 4,000 ×g for 20 min to recover plasma. The plasma samples were collected using dry Pasteur pipettes, and stored at −80°C until further use. Using enzyme-linked immunosorbent assay (ELISA), plasma cytokine profile including reactive oxygen species (ROS), IL-6, and C reactive protein (CRP) were determined as previously described (37, 38), while caspase-3 and caspase-9 levels were measured (39) using commercially available kits (R&D Systems, USA) and according to the manufacturer’s instructions. Plasma samples were also analyzed using commercial kit (Biodiagnostic, Egypt) for total antioxidant capacity (TAC) according to the instructions of the manufacturer. Absorbance was measured with a spectrophotometer at wave length of 505 nm. Peripheral blood mononuclear cells (PBMCs) were isolated from the cellular components of the blood after plasma isolation using Ficoll-Paque method. Whole-cell lysates were prepared from PBMCs isolated from the three animal groups using RIPA buffer. Following centrifugation at 16,000 g for 15 min at 4°C, the protein concentration of each supernatant was determined using a protein assay kit (Bio-Rad, Hercules, CA) as previously described (40, 41).

***Western blotting***

Seventy milligrams of each whole-cell protein lysates were separated by discontinuous SDS-PAGE. The proteins were then transferred onto nitrocellulose membranes, which were blocked for 1 hr at room temperature with 1% bovine serum albumin (BSA) dissolved in Tris-buffered saline (TBS; 20 mM Tris-HCl, pH 7.4, and 150 mM NaCl) supplemented with 0.1% Tween 20. The membranes were then incubated in the same blocking buffer containing primary antibodies against Bcl-2, Bcl-2-interacting mediator of cell death (Bim and Bax), survivin, Cytochrome C, and β-actin (1:1,000; SantaCruz Biotechnology). The blots were washed and then incubated with an HRP-labeled species-matched secondary antibody for 1 hr at room temperature. Protein bands on the membranes were detected by enhanced chemiluminescence (ECL, SuperSignal West Pico Chemiluminescent Substrate, Perbio, Bezons, France). The ECL signals were recorded on Hyperfilm ECL. To quantify the protein band intensities, the films were scanned, saved as TIFF files, and analyzed using NIH ImageJ software as previously described (42).

***Immunohistochemical analysis***

Paraffin-embedded sections of spleen and thymus from the three animal groups were fixed in a freshly prepared formal alcohol as previously described (34). Tissue sections were stained using primary antibody (anti-HSP-70). Tissue sections were cleared in xylene, rehydrated in graded ethanol (100%–70%), and immersed in distilled water for 5 min. Epitopes were unmasked by boiling sections in citrate buffer (pH 6) for 40 min. After this antigen retrieval step, sections were washed twice in phosphate-buffered saline (PBS). For inhibition of endogenous peroxidase activity, sections were incubated in 0.5% hydrogen peroxide dissolved in PBS for 7–15 min and were then washed with PBS before incubation with the primary antibody (anti-HSP-70; Santa Cruz Biotechnology, USA) diluted 1:100 for thymus and 1:500 for spleen sections for 30 min. Sections were then washed with PBS and incubated for 30 min with peroxidase labelled secondary antibody (KPL, USA) diluted 1:200. Finally, sections were visualized using DAB-chromogen (Dako, Denmark) for 2–5 min and counterstained with Mayer hematoxylin (Dako, Denmark) for 5 min, then dehydrated and mounted. 

***Electron microscopic study***

Small pieces (1×1 mm) of bone marrow from each group were quickly removed, and fixed in formal alcohol. The specimens were then washed four changes of 15 min each with slow shaking in PBS and then fixed in 1% osmium tetroxide for 2 hr. The samples were then embedded in Epon 812 for 4 hr. Samples were finally embedded into capsules containing the embedding mixture, and the tissue blocks were polymerized in an oven for 2 days at 60 ^°^C. The ultra-thin sections were prepared and were stained with uranyl acetate and lead citrate for examination under transmission electron microscope (TEM).

***Light microscopic study***

For the histological examination of bone marrow, thymus and spleen cut sections were stained with Hematoxylin–Eosin (H&E) according to method described by Drury and Wallington (43). We used 14% EDTA for 6-9 days for bone marrow decalcification.

***Statistical analyses***

Data were tested for normal distribution and were expressed as mean ± standard error of the mean (SEM). Significant differences among animal groups were statistically analyzed by a one-way ANOVA accompanied by Tukey’s *post hoc* test for multiple comparisons, using PRISM statistical software (GraphPad Software). Differences were considered significant at *P*<0.05.

## Results

***CWP supplementation restores elevated levels of IL-6 and ROS induced by HS***

Pro-inflammatory cytokines such as IL-6 are involved in the response to HS, leading to induction of free radicals and oxidative stress, which suppress immune function. Using ELISA, we measured IL-6 and ROS levels in the plasma of mice from control, HS, and CWP-HS groups. We found that HS mice exhibited higher levels of IL-6 and ROS compared to control animals, whereas CWP-HS mice exhibited significantly lower levels of IL-6 and ROS as compared to HS animals (Table 1).

***Treatment with CWP enhances*** ***TAC level while decreases CRP levels***

CRP plasma concentration increases during inflammation as it participates in the systemic response to inflammation. To evaluate the antioxidant defense of CWP against HS, we measured TAC in the plasma. We measured the levels of CRP and TAC in the plasma of mice from control, HS, and CWP-HS groups. Accumulated data revealed that the levels of CRP (Figure 1A) were significantly increased in HS mice compared to control animals. In contrast, CWP-HS mice exhibited a significant reduction in CRP level compared to HS group. Moreover, HS mice exhibited a marked decrease of TAC level compared to control mice (Figure 1B). In contrast, CWP supplementation significantly restored TAC level compared to untreated HS mice.

***Oral supplementation of CWP in HS mice restores expression of survivin, Bcl-2 family members, and Cytochrome C***

We next investigated the potential effect of CWP treatment and the underlying mechanism of apoptosis in mice after exposure to HS. Cells were collected from 3 individual mice from control, HS, and CWP-HS groups and were lysed using lysis buffer. Western blot analysis was used to analyze the expression of cell cycle regulatory protein (survivin), the expression of some members of Bcl-2 family (including the anti-apoptotic member Bcl-2 and the pro-apoptotic members Bax and Bim), and the expression of cytochrome C. As shown in (Figure 2A), the results of one representative experiment demonstrated that treatment with CWP significantly increased the expression of survivin and the anti-apoptotic Bcl-2 protein compared to HS group. By contrast, treatment with CWP clearly decreased the expression of the pro-apoptotic Bax and Bim, and the expression of cytochrome C compared to HS group. The expression levels of the analyzed proteins were normalized to total β–actin protein levels. The accumulated data from five mice in each group (represented in Figure 2B, C) clearly illustrated that treatment with CWP significantly increased (*P*<0.05) the expression of the anti-apoptotic Bcl-2 protein and survivin compared to untreated HS group. By contrast, CWP treatment significantly decrease (*P*<0.05) the expression of Bax and Bim, and cytochrome C, respectively (Figure 2D, E, F). Interestingly, our data revealed that the treatment with CWP increased expression of survivin and the anti-apoptotic Bcl-2 protein, which was accomplished by a decrease in the expression of Bax, Bim and cytochrome C that participate in the apoptosis process, which all together inhibit apoptosis induced by exposure to HS.

***Anti-apoptotic effect of CWP against HS via modulating caspases pathway***

Since effectors involved in caspase cascades are key mediators of apoptosis induction, using a fluorometric protease assay we monitored the activity of caspase-3 and caspase-9. The measured data of 5 mice from each group showed that treatment with CWP significantly decreased the activity of caspase-3 and caspase-9 compared to untreated HS mice (Figure 3 A, B). 

***CWP improves histological and ultrastructure alterations of bone marrow induced by exposure to HS***

We evaluated the effect of HS and CWP on the histological architecture and ultrastructure of bone marrow. In the control group (Figure 4A), normal histological features of vascular sinuses and different hematopoietic cells (lymphoid cells, myeloid cells, and megakaryocyte cells) were observed. In HS group (Figure 4B), a wide dilation and thrombosis in vascular sinuses with discontinuous endothelial cells lining was observed. While in CWP-HS group (Figure 4C), animals exhibited partial restoration in the architectures of bone marrow similar to those of the control animals. Ultrastructure examination using TEM of control group (Figure 4D) showed different hematopoietic cells such as monocyte, neutrophil, eosinophil and lymphocyte cells. In HS animals (Figure 4E), shrunken cells with degenerated cytoplasm, together with numerous signs of apoptotic cells were observed, which are characterized by electron dense pyknotic nuclei. Presence of apoptotic bodies of nucleus (karyorrhexis) were noticed as well. While in CWP-HS animals (Figure 4F), a partial improvement was observed by the restoration of normal appearance of nuclei shape with centrally located euchromatin and heterochromtin aggregates at the periphery of islands, and decreased number of apoptotic cells. 

***CWP supplementation improves altered distribution of HSP-70 induced by HS in the thymus***

We investigated the effect of HS and CWP on the histological architecture and distribution of HSP-70 in the thymus as a primary lymphoid organ. In Figure 5A, thymus section of the control group showed the normal histological structure of cortex and medulla, while the cortex of heat stressed animals was characterized by the appearance of congested blood vessel with depletion of thymocytes (Figure 5B). In contrast, CWP-HS animals nearly restored the normal histological structure of cortex and medulla of the thymus (Figure 5C). Anti-HSP-70 primary antibody was used to detect the presence of HSP-70, which is expressed in the cytoplasm and nucleus. In control group (Figure 5D), animals exhibited HSP-70 expressing lymphocytes that were moderately distributed in the medulla and were quietly scattered in the cortex, while HS animals (Figure 5E) revealed a marked increase of HSP-70 expressing lymphocytes in the medulla and cortex. In CWP-HS animals (Figure 5F), the distribution of HSP-70 expressing lymphocytes in the medulla and cortex was partially similar to that in control group.

***Treatment with CWP ameliorates splenic architecture and distribution of HSP-70 secreting cells***

We investigated the effect of HS and CWP on the histological architecture and distribution of HSP-70 in the thymus as a secondary lymphoid organ. Figure 6A shows spleen section from control group with normal morphology of red and white pulps. In HS animals (Figure 6B), spleen section revealed focal area of lymphocytic necrosis and depletion of white pulp surrounded by red pulp vascular congestion. In contrast, CWP-HS animals (Figure 6C) exhibited a partial restoration to the normal histological splenic architecture of the white and red pulps. In general, HSP-70 was detected in the cytoplasm and nucleus of cells that present in the active germinal center, which produces lymphocytes after antigen stimulation. Figure 6D showed control spleen with normal distribution of HSP-70 secreting lymphocytes in the periarterial lymphatic sheath. In HS animals (Figure 6E), there was a marked increase in the expression of HSP-70 in the periarterial lymphatic sheath of spleen. While in the CWP-HS mice (Figure 6F), the distribution of HSP-70 secreting lymphocytes was almost similar to that in control group.

## Discussion

Oxidative stress contributes to many human diseases, and natural antioxidants are involved in enhancing immune function via oxidative stress-dependent mechanisms. For example, we previously demonstrated the ameliorating impact of natural antioxidants isolated from snake venom, which improved normal lymphocyte functions and exerted anticancer effects through reduction of oxidative stress in various human and animal cells (35, 38, 39, 44, 45). It has been proven that CWP has immunomodulatory role through stimulating the chemotaxis of different immune cells towards different chemokines in mice model (16). Therefore, the focus of studies to investigate the impact of antioxidants in food has increased, particularly the effects of milk and milk-derived peptides, which are widely consumed daily by humans, on public health and immune system function (46). 

In the current study, we found that HS induced the levels of ROS, which in turn increases the free radicals and oxidative stress in HS animals. These findings are in agreement with other study, which reported that HS induced ROS production (47). In addition, treatments of HS animals with CWP significantly decreased ROS levels, indicating that CWP may improve oxidative stress caused by exposure to HS. In accordance with our findings, the antioxidant activity of CWP could be explained as it acts as a chelating agent on toxicants (48), and camel milk is reported to contain high levels of minerals that act as free radical scavengers (49). Our current data showed that HS increased IL-6 level as a pro-inflammatory cytokine, which is in agreement with a study reporting that the levels of pro-inflammatory cytokines such as IL-6, TNF-α and IL-1β can be elevated in animals exposed to HS (50). When HS mice were treated with CWP, they had restored IL-6 level due to anti-inflammatory effect of CWP.

CRP is secreted from liver in response to inflammatory cytokines such as IL-6 (51). It has been reported that CRP decreases due to consumption of diets containing fruits and vegetables as a rich source of flavonoids antioxidants (52). This could explain why HS elevated CRP level, while CWP significantly restored it to the normal value. TAC could determine the whole content of antioxidants in the body as it can detect all the antioxidants found in plasma or body fluids. Therefore, it provides an accurate evaluation of the physiological, environmental and nutritional factors of animal (53). Additionally, it was previously shown that heat stroke rats displayed decreased cardiac TAC (54). This is in accordance with our results where HS caused inhibition in TAC level, meanwhile when HS animals were treated with CWP, we found significant restoration of TAC to control level. 

Overproduction of ROS causes disruption of mitochondrial membrane and the release of cytochrome C (55). When cytochrome C enters the cytosol, it binds to apoptosomes thus initiating the caspase cascade leading to caspase-3 activation (56). It was proven that HS causes loss of mitochondrial membrane potential thus release of cytochrome C from mitochondria as well as overproducing ROS (57). Additionally, it has been shown that HS caused elevation of Apaf 1, caspase-9 and caspase-3 levels, and DNA fragmentation (58). This is in agreement with our data that HS increased caspase-9 and caspase-3 due to apoptosis, while CWP significantly restored these levels due to its anti-apoptotic role. Anti-apoptotic Bcl-2 protein inhibits apoptosis through preventing the release of cytochrome C from mitochondria. HS activates Bax and Bim as pro-apoptotic proteins and increases mitochondrial outer membrane permeability leading to release of cytochrome C (59). This agrees with our results that HS induces apoptosis through significantly upregulating Bax and Bim expression, while significantly downregulating Bcl-2 expression. Our results are in accordance with another study demonstrating that CWP restored the ratio of Bax/Bcl-2 near to the normal ratio (60).

Survivin is one member of apoptosis inhibitor proteins family, which is expressed during the cell division in cell cycle (61). It contributes to monitoring of cell division and suppression of apoptosis (62). In cell cultures studies, survivin overexpression was associated with inhibition of apoptosis through intrinsic or extrinsic apoptotic pathways (63-65). This would explain the downregulation of survivin in the HS mice, while its level restored significantly to the normal value by treatment with CWP.

It has been reported that only HSP-70 was obviously induced in immune cells after heat preconditioning (66). Lymphocytes were suggested to be a specific releasers among immune cells to secrete 100% of total extracellular HSP-70 (67). We identified the lymphocytes as the immune cells that were positive to immunohistochemical staining of HSP-70 in spleen and thymus. It has been shown that fever-range temperatures enhanced HSP-70 expression in spleen cells. Additionally, HSP overproducing immune cells have a better ability to overcome physical or chemical stressors (68). This explains the overproduction of HSP-70 secreting lymphocytes in the spleen of the HS animals, while this increase was restored nearly as in control by treatment with CWP.

HSP-70 is able to distinguish whether to repair the cellular damage and inhibit apoptosis or allow apoptosis to occur when the cellular damage is untreatable. It has been declared that the HSP-70 role in HS-inducing apoptosis could be either suppressing apoptosis or allowing cell death without interfering in the apoptotic pathway (69) thus preventing DNA mutations caused by the stress (70). Although thymocytes are highly producing HSP-70, they undergo excessive apoptosis, because hyperthermia causes acceleration of normal thymus cellular division and differentiation, leading to increased apoptosis of immature thymocytes (71). This is in accordance with our results that HSP-70 secreting thymocytes increased in the medulla of the HS animals more than the cortex, while it was restored nearly normal value in the CWP-treated HS mice.

When apoptosis elements enter nucleus, they cause condensation and fragmentation of chromatin (72). This may explain the severe ultrastructure destruction appeared in TEM of bone marrow in our HS animals. As we found shrunken cells with degenerated cytoplasm, numerous signs of apoptotic cells were also observed, which are characterized by electron dense pyknotic nuclei. Oxidative stress produces tissue injury through apoptosis and necrosis (73). Additionally, it was reported that HS caused vascular endothelium injury, as the endothelial cells are the early affected cells during HS (74, 75). These findings may explain the histopathological changes of lymphoid organs induced by HS in our results. As shown in H&E staining of bone marrow sections of HS mice, we found wide dilation and thrombosis in vascular sinuses with discontinuous endothelial cells lining. Additionally in H&E staining of thymus sections of HS mice, there was congested blood vessel with depletion of thymocytes in the cortex. Finally, in H&E staining of spleen sections of HS mice, we found focal area of lymphocytic necrosis and depletion of white pulp surrounded by red pulp vascular congestion. It was previously declared the enhancing effects of CWP on the histological architecture of the immune organs (spleen, thymus and bone marrow)-induced damage due to type 1 diabetes (60). This is in accordance with our results that CWP improved the harmful pathological and ultrastructure changes in the CWP-treated HS group. As demonstrated in H&E staining sections of bone marrow, thymus and spleen of CWP-HS mice, there were partial restoration of the architectures of these tissues nearly to the control normal histology.

## Conclusion

Our study revealed that CWP supplementation of mice exposed to HS has anti-hyperthermic effect via decreasing ROS and enhancing TAC thus improving the redox status. CWP also regulated the Bax and Bim ratio to Bcl-2 expression thus ameliorating the pathological alterations that occur due to apoptosis induced by HS. For the first time, our current study proved that CWP suppressed apoptosis through direct restoration of apoptotic elements; cytochrome C, caspase-9 and caspase-3. Anti-inflammatory role of CWP was obvious to suppress IL-6 and CRP. The result of present study showed for the first time that CWP regulated cell cycle through modulating survivin expression near to normal value. These data revealed the beneficial effects of CWP supplementation in improving architectures and functions of lymphoid organs. 

## Conflicts of Interest

The authors declare that they have no competing interests.
